# Quantifying the clonality and dynamics of the within-host HIV-1 latent reservoir

**DOI:** 10.1093/ve/veaa104

**Published:** 2021-01-06

**Authors:** Roux-Cil Ferreira, Jessica L Prodger, Andrew D Redd, Art F Y Poon

**Affiliations:** 1 Department of Pathology and Laboratory Medicine, Western University, 1151 Richmond Street London, ON, Canada; 2 Department of Microbiology and Immunology, Western University, 1151 Richmond Street London, ON, Canada; 3 Laboratory of Immunoregulation, National Institute of Allergy and Infectious Diseases, National Institutes of Health, 5640 Fishers Lane Rockville, MD 20852, USA; 4 Department of Medicine, Johns Hopkins School of Medicine, 600 N. Wolfe Street Baltimore, MD 21205-2196, USA

**Keywords:** HIV-1 latency, clonality, within-host evolution, branching processes

## Abstract

Among people living with human immunodeficiency virus type 1 (HIV-1), the long-term persistence of a population of cells carrying transcriptionally silent integrated viral DNA (provirus) remains the primary barrier to developing an effective cure. Ongoing cell division via proliferation is generally considered to be the driving force behind the persistence of this latent HIV-1 reservoir. The contribution of this mechanism (clonal expansion) is supported by the observation that proviral sequences sampled from the reservoir are often identical. This outcome is quantified as the ‘clonality’ of the sample population, e.g. the fraction of provirus sequences observed more than once. However, clonality as a quantitative measure is inconsistently defined and its statistical properties are not well understood. In this Reflections article, we use mathematical and phylogenetic frameworks to formally examine the inherent problems of using clonality to characterize the dynamics and proviral composition of the reservoir. We describe how clonality is not adequate for this task due to the inherent complexity of how infected cells are ‘labeled’ by proviral sequences—the outcome of a sampling process from the evolutionary history of active viral replication before treatment—as well as variation in cell birth and death rates among lineages and over time. Lastly, we outline potential directions in statistical and phylogenetic research to address these issues.

## 1. Introduction

Integration of viral complementary DNA into the host genome is an obligate step in the human immunodeficiency virus type 1 (HIV-1) replication cycle (Lewinski and Bushman 2005). Once integrated, viral genes can be expressed by the host cell machinery. Cells that actively express the HIV-1 proviral DNA are relatively short-lived due to cytotoxicity induced by viral components (Pollack et al. 2017) and targeting of the infected cells by the adaptive immune response (Migueles and Connors 2015). On the other hand, integration of HIV-1 DNA into resting CD4+ T cells, or those about to transition to a resting state, establishes a long-lived population of infected cells known as the latent viral reservoir that is established early in infection and continually supplemented throughout viremic disease (Chavez, Calvanese, and Verdin 2015). Latently-infected cells have little to no transcription of integrated HIV-1 DNA, making the infected cell virtually invisible to the adaptive immune response. In addition, current antiretroviral treatment (ART) does not target the integrated provirus in these cells. Reactivation of infected cells from the latent reservoir appears to occur at a predictable rate, such that the virus population is quickly reseeded to pre-therapy levels following an interruption in treatment (Chun et al. 2010). Thus, the long-term persistence of the latent viral reservoir is a major obstacle to the complete eradication of the virus from an infected individual, i.e. a sterilizing HIV-1 cure. Understanding the dynamics within the latent reservoir may also become important for determining the extent that the reservoir must be depleted in order to achieve a functional cure, such that immune control of virus replication can be sustained for years in absence of treatment (Davenport et al. 2019). While empirical measurements of reservoir decay rates (Crooks et al. 2015) may be pessimistic due to stochastic effects once the reservoir gets small (Conway and Coombs 2011), they indicate that the latent reservoir will almost surely persist throughout the expected lifespan of individuals despite effective long-term suppression of viral replication by ART. Consequently, this population of infected cells is the primary focus for current research in order to design and assess potential HIV-1 reactivation and eradication therapies (Katlama et al. 2013).

Since completely suppressive ART inhibits viral replication, the division of infected resting CD4+ T cells is likely the primary contributor to the persistence of the latent reservoir (Murray et al. 2016; Bozzi et al. 2019). Some controversial findings suggest that ongoing virus replication in anatomic compartments with low drug penetrance might also contribute to the persistence of the latent reservoir (Lorenzo-Redondo et al. 2016; Nolan et al. 2018). However, subsequent studies have reported evidence that ongoing low-level virus replication is most likely not occurring (Van Zyl et al. 2017; Bozzi et al. 2019). The processes shaping viral populations in the latent reservoir are complex and incompletely understood. In addition, these processes may be affected by a number of viral and host factors and their interactions; for example, the infected CD4+ T-cell phenotype (Lee et al. 2017), its antigen-specificity (Simonetti et al. 2016), the integration site of HIV-1 (Maldarelli et al. 2014; Haworth et al. 2018), etc. Despite our growing understanding of the nature of the latent reservoir, many questions remain about its composition and the ability of current methods to measure and characterize it accurately.

Here, we examine the quantitative methods used to summarize sequence data of the latent viral reservoir produced by various assays. We consider the adequacy of the current quantitative measurements of clonality as summary statistics for hypothesis testing, as opposed to taking the occurrence of identical sequences as sufficient evidence of clonal expansion. We further argue that, while determining the existence of clonal expansion is important and that some questions remain regarding this hypothesis, the primary goal is generally to assess the contribution of clonal expansion to reservoir persistence. For this reason, the effect size of clonality on the long-term persistence of the latent reservoir should be explored and directly calculated—this should be done by placing the problem in the context of phylogenetic and statistical inference. Lastly, we highlight factors that may impact the accuracy of these methods, which would ultimately influence the conclusions that can be supported from latent viral reservoir sequence data.

## 2. Sequencing the latent reservoir

### 2.1 Assays measuring the latent reservoir

There are several different approaches used to sample the genetic diversity of integrated HIV-1 lineages within a host, which can be broadly categorized as sequencing the proviral DNA, the associated integration sites, or viral RNA produced from re-activated cells *in vitro* using viral outgrowth-based methods. Proviruses can be sequenced in large numbers from DNA extracted from infected cells using primers targeting relatively conserved regions of the virus genome (Salminen et al. 1995). However, many of the sequences archived in the reservoir represent defective proviruses that are no longer replication-competent due to the introduction of mutations into the genome, including large deletions (Bruner et al. 2016) and hypermutation induced by host factors (Sadler et al. 2010). Some of these proviral sequences can be filtered on the basis of extreme mutations like frameshift-inducing indels, but it is not feasible to exclude all defective proviruses based on their genetic composition alone, due to sequencing constraints and since the impacts of some mutations on viral fitness cannot be predicted accurately. Furthermore, random variation from sequencing error will tend to inflate the observed number of proviral sequence variants. Thus, proviral sequence data will inevitably overestimate the size of the latent reservoir, which is conventionally defined as the replication competent subset of the latently-infected cell population (Wang et al. 2018b). In contrast, methods that target HIV-1 integration sites sequence both the long terminal repeat of the provirus and the flanking sequence in the host genome (Schröder et al. 2002). Because coverage of the provirus genome is limited, it is more difficult to identify defective proviruses from sequences targeting integration site junctions (Maldarelli et al. 2014). On the other hand, integration sites uniquely label proviruses that descend from different integration events; we will expand on this feature in a subsequent section.

Viral outgrowth assays (VOAs) use a limiting dilution method (Taswell 1981) to estimate the size of the latent reservoir. The presence or absence of infected resting CD4+ T cells at a given dilution is determined by co-culturing the sample with other cells that are susceptible to HIV-1 infection, which amplifies the viral outgrowth from any infected cells in the culture well to detectable levels. With some exceptions (e.g. all negative or all positive outcomes), culturing replicates at a series of dilution factors provides sufficient information to estimate the number of infected cells per blood volume (Laird et al. 2016), often denoted as the infectious units per million cells. In addition, sequencing the HIV-1 RNA from VOAs can provide information on the genetic composition of the replication-competent reservoir. VOAs are generally labor-intensive experiments because several replicates at different dilution levels must be cultured for weeks in the lab. On the other hand, VOAs have been characterized as the ‘gold standard’ for quantifying the latent reservoir because the assay only detects cells containing replication-competent provirus. VOA-based studies have been used to demonstrate that the latent reservoir can persist for years without producing virus while retaining the ability to do so after stimulation (Siliciano et al. 2003). However, not all cells that contain intact provirus are stimulated to produce virus *in vitro*, even with multiple rounds of stimulation (Hosmane et al. 2017). For this reason, VOAs are expected to underestimate the size of the latent reservoir.

### 2.2 Clonality of the latent reservoir

The composition and dynamics of the latent reservoir is inferred from the observed frequencies of genetic sequence variants. The division of cells carrying integrated HIV-1 DNA will increase the probability of sampling provirus with identical genetic sequences, because the mutation rate of the human genome is orders of magnitude lower than the mutation rate of the actively replicating virus (Cuevas et al. 2015). The occurrence of identical sequences is referred to as the ‘clonality’ of a sample. Thus, higher levels of clonality provide evidence that the proliferation of infected resting CD4+ T cells plays an important role in the long-term persistence of the latent reservoir (Joos et al. 2008; Hosmane et al. 2017).

As stated above, a key advantage of integration site analysis is that the provenance of each provirus is unambiguous. Sequencing the flanking host genome enables investigators to map the provirus integration site. Given the number of potential sites in the human genome (roughly 3 billion nucleotides), the probability that two proviruses that resulted from separate integration events share the same integration site by chance is exceedingly small (Maldarelli et al. 2014). Therefore, observing two or more sequences from the latent reservoir with the same integration site implies that they are related through clonal expansion, i.e. they are descendants of the same integration event. In this method, the composition of the HIV-1 DNA covered by each sequence does not affect its assignment to different clonal variants, although as stated earlier the replication capacity of the provirus is not known.

In contrast, proviral and VOA-based sequencing methods typically cover a limited interval of the HIV-1 genome (about 2,000 bp) to characterize the genetic composition of the reservoir. The probability that two distinct proviruses are misclassified as instances of the same clonal variant—because their genetic differences fall outside of the sequenced region—is not negligible. This limitation was recognized by Laskey et al. (2016) who developed an empirical weighting scheme, denoted the ‘clonal prediction score’, to identify optimal sequencing targets in the HIV-1 genome. The clonal prediction score was derived from alignments of near full-length HIV-1 genome sequences (e.g. not including the *nef* accessory gene) that were sampled from the same host. Assuming these alignments are representative of variation in the respective reservoirs, they quantified the empirical probability that two identical sequences spanning a given interval of the HIV-1 genome would have genetic differences outside the sequenced region. For short reads (100–500 bp), less than 60 per cent of sequences on average were incorrectly classified as identical copies of the same variant. Using longer reads (6 kbp) reduced the chance that differences were located outside of the sequenced region, such that fewer than 20 per cent of sequences on average were misclassified as being clonal (Laskey et al. 2016).

A related but distinct problem of interpreting sequences from the latent reservoir is that genetically identical proviruses may be the result of separate integration events. This ‘collision’ of proviral sequence labels is more likely to occur if the actively replicating virus population was predominantly genetically homogeneous when these viruses were deposited into the latent reservoir. For example, the majority of infections originate from a single transmitted founder virus followed by a period of exponential growth (Keele et al. 2008; Joseph et al. 2015). Suboptimal ART can also provide opportunities for the virus population to evolve drug resistance, which may induce a ‘hard’ selective sweep in which the right combination of mutations arises in a single genetic variant (Feder et al. 2016). Studies of the genetic composition of the latent reservoir tend to focus on subjects who initiated treatment at a chronic stage of infection. If most cells in the latent reservoir carry provirus that became integrated around the time of treatment initiation (Brodin et al. 2016; Abrahams et al. 2019), then the probability of label collision should be low. However, it is generally believed that not all integration events map to treatment initiation, and that provirus may be deposited into the reservoir throughout the course of untreated infection (Jones et al. 2018; Pankau et al. 2020). Thus, the ability to estimate the chance of collisions in proviral sequence labels is contingent on furthering our understanding of the dynamics of reservoir formation.

In conclusion, resolving a clear picture of the reservoir from the analysis of sequence variation remains an open question. Thus, developing and improving both experimental assays and the analyses applied to these sequence data is key to better understanding the reservoir.

## 3. Analyzing reservoir sequences

The current standard approach for quantifying the contribution of clonal expansion in the reservoir is to report either (i) the proportion of sequences that are identical to one or more other sequences in the sample (Wagner et al. 2014; Von Stockenstrom et al. 2015; Lorenzi et al. 2016; Hosmane et al. 2017; Lee et al. 2017; Satou et al. 2017; Salantes et al. 2018; Salantes et al. 2018), i.e. multiple instances of the same sequence ‘variant’; or (ii) the proportion of sequence variants that are observed more than once in the sample (Maldarelli et al. 2014; Wagner et al. 2014; Haworth et al. 2018). To illustrate, suppose that we have sequenced provirus form seven latently infected cells. Three of the variants share one identical sequence; similarly two others are also identical, and the remaining two variants have unique sequences. In sum, four unique sequence variants are observed 3, 2, 1, and 1 times, respectively. Depending on whether we use the number of sequences or the number of variants as the denominator, one would report the clonality as either 5/7 (71%) or 2/4 (50%).

These quantities do not provide any means of evaluating whether there is *statistically significant* clonality in the sample. Therefore, we need to introduce more formal mathematical notation. Let *N*(*t*) and *V*(*t*) be the total number of reservoir cells and the total number of distinct sequence variants in the reservoir at time *t*, respectively. For now, we will set aside the issue of whether sequences are correctly assigned to variants. Suppose that we sample *n* sequences, $S=\{s_{1},\ldots ,s_{n}\}$, at time *t* where n<N(t). Let *P* be the partition of *S* into a finite number of non-empty subsets $v_{i}\in P$ that correspond to different variants in the sample indexed by *i*. We allow for some variants to fail to appear in the sample such that ||P||≤V(t). Each subset *v_i_* has *n_i_* elements that represent the abundance of each sequence variant in the sample, such that $n=||S||=\sum_{i}n_{i}=\sum_{i}||v_{i}||$. The two conventional summary statistics for measuring clonality are therefore:

$$p_{1}=\frac{\sum_{v_{i}\in P}n_{i}\;I(n_{i}>1)}{\left||S|\right|}\;\text{and}\;p_{2}=\frac{\sum_{v_{i}\in P}I(n_{i}>1)}{\left||P|\right|}$$

where *I*(*x*) is an indicator function that takes the value 1 if *x* is true and 0 otherwise, and ||x|| is the number of elements in *x*. As per the example above, we observed the partition $\left\{v_{1}=\{s_{1},s_{2},s_{3}\},v_{2}=\{s_{4},s_{5}\},v_{3}=\{s_{6}\},v_{4}=\{s_{7}\}\right\}$, then $p_{1}=5/7$ and $p_{2}=2/4$.

These summary statistics (*p*_1_ and *p*_2_) have been used to demonstrate that large proportions of sequences sampled from the reservoir tend to be members of one or more clonal populations (Bui et al. 2017; Hosmane et al. 2017; Salantes et al. 2018). However, we do not understand the sampling properties of either statistic. For instance, because we can only work with incomplete samples of the latent reservoir, there is a reasonable chance that a variant present in substantial numbers in the reservoir is present in a single copy (i.e. a ‘singleton’) in the sample. By dichotomizing variants into clones and singletons, *p*_1_ and *p*_2_ discard a considerable amount of information about the underlying sample abundance distribution (*n_i_*). Thus these statistics are often reported with genealogical trees that not only visualize the common ancestry relating sampled variants, but also their relative abundance as polytomies of varying size (e.g. Hosmane et al. 2017; Lee et al. 2017; Salantes et al. 2018).

**Figure 1.**
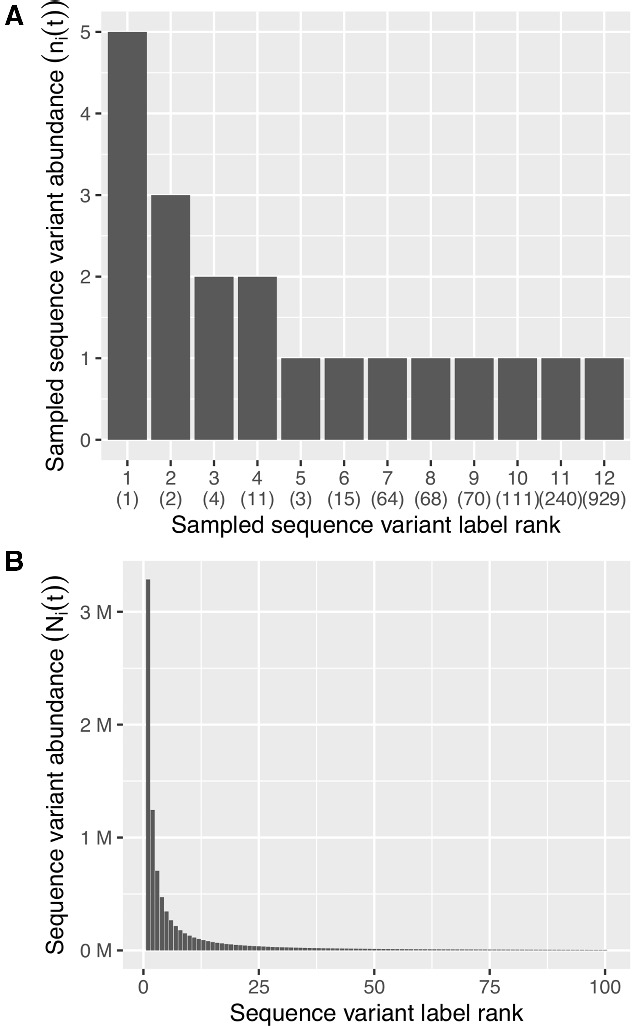
Sampling properties under a power-law model. (A) An example of an ordered histogram of *n *=* *20 sequences sampled from a hypothetical reservoir population represented in (B). The true rank in the sequence population is displayed in brackets. (B) The power-law distribution of rank abundance for α=1.4 (replication-competent estimates in Reeves et al. (2018)) in a reservoir of 10^7^ cells comprising 10^4^ variants (only the first 100 ranks are displayed).

Improving on *p*_1_ or *p*_2_ requires that we employ some parametric model to estimate the underlying number of distinct sequence variants in the reservoir, *V*(*t*), which is not a trivial task even when we disregard labeling errors, i.e. variant misclassification. For example, Reeves et al. (2018) fit a power law model $\left(x^{-\alpha }\right)$ to the rank abundance distribution of sequence variants sampled from the latent reservoir (Figure 1). Briefly, the rank abundance is an ordered histogram of the sequence variants, *v_i_*, such that the vertical axis (counts) represents the sampled abundance $\left(n_{i}\right)$ of each sequence variant, and the sequence variant in the first position, *v*_1_, has the highest abundance ($v_{1}=n_{1}>n_{j}\;\forall j>1$, Figure 1A). If the reservoir population, *N*(*t*), is distributed among the sequence variants, *V*(*t*) according to a power law model, $N(r)\propto r^{-\alpha }$, then the reservoir would comprise mostly of the clones of a small number of sequence variants (∼80%, i.e. the Pareto principle; Figure 1B). However, there would also be many singletons and sequence variants with comparatively low abundance. To estimate the total number of variants *V*(*t*) and the power-law exponent *α*, Reeves et al. (2018) fit this model to sequence data from two different sources: first to data from a VOA study, and subsequently to data from two integration site assay studies. *N*(*t*) was fixed to values obtained from the literature for each data source. The resulting estimates of *V*(*t*), *α* and the reservoir distribution across sequence variants conveyed several implications regarding the composition of the reservoir.

Firstly, the proportional statistics *p*_1_ and *p*_2_ systematically underestimate the true extent of clonal expansion, due to incomplete sampling of the reservoir (Reeves et al. 2018). The effect of incomplete sampling manifests as a consequence of the presumed underlying distribution of abundance across the sequence variants in the reservoir population, i.e. a small sub-population of sequence variants represent a large fraction of the total reservoir. This distribution implies that at the start of sampling, additional samples uncover new sequence variants. However, as sampling progresses we experience diminishing returns and encountering new variants becomes increasingly rare, since additional samples will be dominated by the sequence variants representing the highly abundant sub-population. Furthermore, due to the skewed abundance distribution, the true diversity of the reservoir *V*(*t*) will be very difficult to measure using sequence data alone, even if a considerable increase in sampling effort (100-fold increase) was undertaken (Reeves et al. 2018). Lastly, they postulated that differences between estimates obtained from VOA versus integration site data suggested that a smaller number of extremely abundant variants may make up a greater proportion of the replication-competent reservoir when compared to all integrated HIV-1, i.e. the distribution in Figure 1B representing the replication-competent sequence variants is skewed to the left to a greater extent than the equivalent distribution for all integrated sequence variants. Whether or not this discrepancy in the estimates is a consequence of differences in the sequencing assays or an accurate representation of the reservoir requires further investigation. For example, Lorenzi et al. (2016) demonstrated that there is poor agreement between outgrowth-, proviral-, and bulk culture assay-based estimates of $\left\{n_{i}\right\}$ for a given individual. While this discordance may predominantly be due to the replication incompetence of large proportions of integrated HIV-1 DNA as hypothesized by Lorenzi et al. (2016), other factors may affect our ability to reactivate intact proviruses *in vitro* such as the integration site or the host cell dynamics.

The methodology employed by Reeves et al. (2018) to gain the above mentioned results had specific limitations which included: the assumption that rank-abundance data are continuous; the fitting of a power-law model to these data; and the statistical robustness of subsequent extrapolation of this model to gain estimates of *N*(*t*) and *V*(*t*). In the following sections we will focus on the general limitations faced by their approach as well as by studies employing *p*_1_ and *p*_2_. Specifically, these approaches do not explicitly evaluate the contribution of cell proliferation (i.e. clonal expansion) to persistence, the role of variation in proliferation among sub-populations in the reservoir, nor the potential heterogeneity of proliferation rates over time.

### 3.1 Proliferation hypothesis

Clonal expansion studies are underpinned by the hypothesis that proliferation of latently infected cells contributes substantially to the persistence of the reservoir. However, neither a quantification of the extent of the proposed contribution (absolute or relative), nor an exact definition of persistence is provided. Here, we will endeavor to specify exactly this hypothesis in mathematical notation.

As before, let *N*(*t*) and *V*(*t*) be the total numbers of infected cells and sequence variants in the reservoir at time *t*, respectively. We define $N_{i}(t)$ to be the number of cells labeled with the *i*-th variant at time *t*, where i∈{1,…,V(t)}, such that:

$$N(t)=\sum_{i=1}^{V(t)}N_{i}(t).$$

This implies that V(t)≤N(t) ∀ t, where *V*(*t*) and *N*(*t*) are equal only if every infected cell is labeled with a unique sequence. Given these working definitions, we can restate the null hypothesis of clonal expansion studies utilizing either *p*_1_ and *p*_2_ as $H_{0}:p_{1}=0\;\text{or}\;p_{2}=0$, or alternatively as:

$$H_{0}:N_{i}(t)=1\;\forall i,t.$$

However, rejecting *H*_0_ does not necessarily relate proliferation to persistence, since this would require making speculative assumptions about the size and dynamics of *N*(*t*) as explored next.

### 3.2 Formalizing persistence

**Figure 2.**
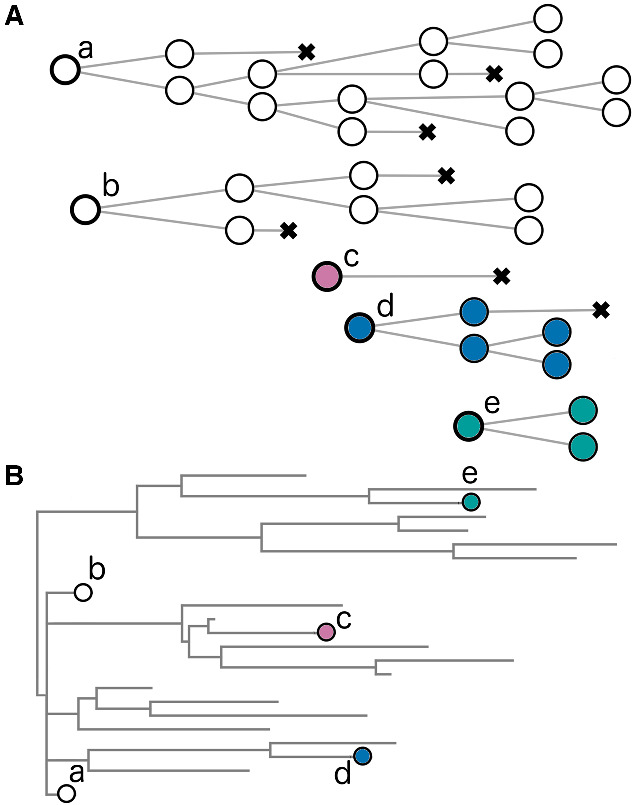
Definition of lineages and labels. (A) Each lineage comprises the initial infected cell (*a–e*, bold outline) that enters a latent state, and its descendants that are also in the latent state. We assume the number of descendants varies over time according to a birth-death process, where a × symbol indicates cell death. Lineages are initiated at different points in time. (B) Lineages initiated at an early stage of infection (*a* and *b*) will tend to carry near-identical labels with respect to the provirus sequence resulting in the ‘collision’ of labels, whereas those initiated at a later stage (*c–e*) will have labels that have accumulated mutations (denoted by colors) during active virus replication before treatment.

Given that persistence is a primary focus in studies of the latent reservoir, we need to formulate a model of reservoir persistence under some simplifying assumptions. First we define a ‘lineage’ as a subset tree (i.e. a contiguous fragment of the original tree) comprising an infected cell that enters a latent state and some of its descendants (Figure 2A). Note that this usage of the term is more similar to lineages as a tier of a viral nomenclature, akin to ‘clades’, rather than a non-branching chain from an ancestor to a single descendant. This definition excludes any descendants that subsequently re-activate and undergo additional rounds of virus replication. As a result, a lineage is permanently ‘labeled’ by the genetic composition of the infecting virus genome and its integration site into the genome of the host cell. In practice, labels are not completely observed; for example, sequencing often covers only a specific part of the provirus genomes (see above). We assume that potential ongoing virus replication in drug sanctuaries, low-level viremia and re-activation of latently infected cells have negligible effects on the composition of the latent reservoir under fully suppressive ART. As per the preceding section, labels deposited into the reservoir at an early stage of infection are expected to be largely homogeneous with respect to proviral sequences (Figure 2B) because of limited diversification in the actively replicating virus population. Under these assumptions, we can conceptually partition the natural history of the infection into a pre- and post-treatment stage. The pre-treatment stage is characterized by rapid expansion and diversification of an actively replicating virus population from which lineage labels are generated. During the post-treatment stage, on the other hand, the identity of labels among the lineages are fixed, since further diversification is halted in the absence of ongoing replication during treatment. Consequently, the overall frequencies of labels in the reservoir are modulated only by the growth and decay dynamics of the respective lineages.

To further simplify the pre-treatment stage of our model, we assume that the clonal expansion of lineages is negligible relative to the rate that new lineages are incorporated into the reservoir prior to treatment initiation. Ignoring growth dynamics in the reservoir pre-therapy enables us to assume that all lineages start at a single copy at treatment initiation—time zero. This approach is supported by recent empirical evidence that the majority of lineages in the reservoir were deposited near the start of treatment (Brodin et al. 2016; Abrahams et al. 2019). Although this assumption restricts the model to lineages that are extant at treatment initiation, it may be possible to reconstruct the actual origin times (integration dates) using a molecular clock (Brodin et al. 2016; Jones et al. 2018). Furthermore, censoring the true abundance of extant lineages at time zero may cause the model to systematically overestimate the lineage proliferation rates following treatment initiation. Given these assumptions we have: $N_{i}(0)=1\;\forall i=1,\ldots ,V(0)$ and $V(0)=N(0)=\sum_{i=1}^{V(0)}N_{i}(0)$. We assume that V(t)≤V(0), which corresponds to the decay of lineages over time.

Therefore, each lineage can be described by an independent discrete branching process with a characteristic rate of birth (through cell division). Following standard branching process theory (Karlin and Taylor 1975), all members of a lineage are independent, live for a single unit of time (non-overlapping generations), produce *Y* offspring and then die. The probability that an individual in lineage *i* produces *y* offspring follows some probability distribution, that is $P(Y_{i}=y)=p_{i}(y)$ with probability generating function $G_{Y_{i}}(s)$. This assumption implies that birth rates are established upon infection of the initial cell, which may be influenced by its CD4+ T-cell phenotype or the integration site of the viral cDNA (Maldarelli et al. 2014). Moreover, some lineages may have higher or lower intrinsic birth rates than others. Thus, the number of individuals at time *t* for lineage *i* can be represented as a randomly stopped sum with a probability generating function $G_{N_{i}(t)}(s)$ that can be expressed in terms of the recursive probability generating function of the lineage-specific offspring distribution $G_{t}^{i}(s)=G_{Y_{i}}(G_{Y_{i}}(\ldots (G_{Y_{i}}(s))\ldots ))$.

In order to define persistence for the whole reservoir population, we first define the probability generating function of the population:

$$\begin{array}{cc} G_{N(t)}(s) & =E[s^{N(t)}]\\ & =E[s^{\sum_{i=1}^{V(0)}N_{i}(t)}]\\ & =E[s^{N_{1}(t)}\ldots s^{N_{V(0)}(t)}]\\ & =\prod_{i=1}^{V(0)}G_{N_{i}(t)}(s)\\ & =\prod_{i=1}^{V(0)}G_{t}^{i}(s) \end{array}$$

The distribution of the exact time of reservoir extinction *T* can now be considered. That is, *T *=* t* if generation t=1,2,… is the first generation with no individuals (Karlin and Taylor 1975):

T=t⇔N(t)=0 and N(t−1)>0

Thus,

$$\begin{array}{cc} P(T=t) & =P\left(N(t)=0\;\cap \;N(t-1)>0\right)\\ & =P\left(N(t)=0\right)-P\left(N(t)=0\;\cap \;N(t-1)=0\right)\\ & =\prod_{i=1}^{V(0)}G_{t}^{i}(0)-\prod_{i=1}^{V(0)}G_{t-1}^{i}(0) \end{array}$$

If we further assume the offspring distributions of each lineage follows a geometric distribution, that is $Y_{i}∼\mathrm{Geometric}(p_{i})$:

$$P(T=t)=\{\begin{array}{cc} \prod_{i=1}^{V(0)}\left(\frac{\Lambda_{i}^{t}-1}{\Lambda_{i}^{t+1}-1}\right)-\prod_{i=1}^{V(0)}\left(\frac{\Lambda_{i}^{t-1}-1}{\Lambda_{i}^{t}-1}\right) & \text{if}\;\Lambda_{i}\neq 1\;\forall i,\\ \prod_{i=1}^{V(0)}\left(\frac{t}{t+1}\right)-\prod_{i=1}^{V(0)}\left(\frac{t-1}{t}\right) & \text{if}\;\Lambda_{i}=1\;\forall i \end{array}$$

where $\Lambda_{i}=E[Y_{i}]=\frac{1-p_{i}}{p_{i}}$. Lastly, if we assume that the offspring distributions of each lineage are identically distributed, i.e. homogeneous proliferation among lineages, with $\Lambda_{i}=\Lambda \neq 1\;\forall i$:

$$P(T=t)={\left(\frac{\Lambda^{t}-1}{\Lambda^{t+1}-1}\right)}^{V(0)}-{\left(\frac{\Lambda^{t-1}-1}{\Lambda^{t}-1}\right)}^{V(0)}.$$

**Figure 3.**
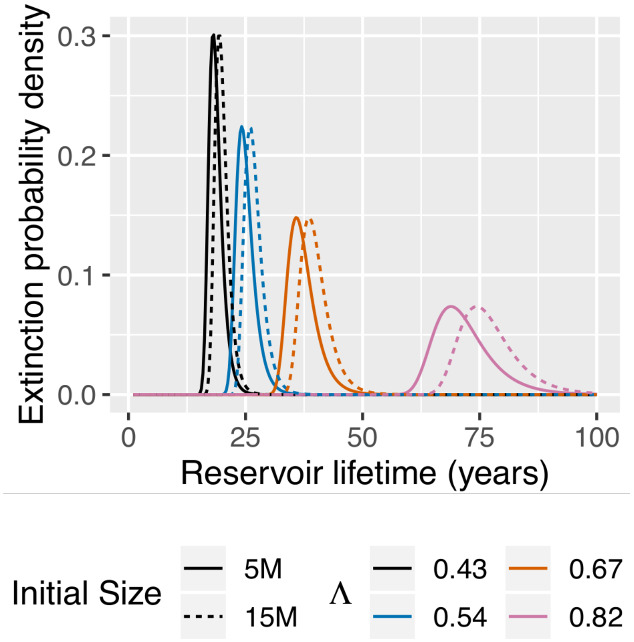
Extinction probabilities of the reservoir over its lifetime. Examples of reservoir extinction distributions with distinct initial reservoir sizes (5 M, solid line; 15 M, dashed line) and Geometric (1/(1+Λ)) offspring distributions, where Λ= 0.43 (black), 0.53 (blue), 0.67 (orange), or 0.82 (pink). Λ represents the expected number of offspring per generation ($\mathrm{yea}r^{-1}$). The half-lives of these example populations are $t_{\frac{1}{2}}\approx$ 0.82 (black), 1.12 (blue), 1.71 (orange), and 3.46 (pink) years.

The preceding equation demonstrates that time to extinction depends on both the initial size of the reservoir, since V(0)=N(0), and proliferation. Moreover, given this formula we can calculate the extinction probability under various conditions (e.g. Figure 3) and determine the impact of proliferation. For example, Conway and Coombs (2011) and Azoz and Coombs (2019) used stochastic continuous-time branching processes to investigate variations in the reservoir extinction probabilities under different model parameter assumptions. The primary goal of the Conway and Coombs (2011) model was to investigate viral blips during ART and, as such, lineage- and time-homogeneous birth and death rates within the reservoir were employed. The Azoz and Coombs (2019) model, on the other hand, used lineage- and time-homogeneous birth rates, but lineage-homogenous time-heterogeneous death rates to investigate the potential impact of latency-reversing drugs on reservoir extinction probabilities. Both of these studies demonstrate the utility of understanding variation in the reservoir extinction times and how these variations are not captured by deterministic models or metric, e.g. half-life estimates or *p*_1_ and *p*_2_.

Some of the assumptions made in the above formulation of the branching process can be relaxed. For example, the proliferation of reservoir cells can occur prior to the initiation of treatment and can be accommodated in the branching process by allowing immigration such that $N(t)=\sum_{i=1}^{V(t)+I(t)}Y_{i}(t+1)$ where *I*(*t*) is the number of new lineages introduced at time *t*, through active virus replication and/or re-integration (see Mitov and Omey 2014). Alternatively, the starting abundance of a lineage can be linked to its ‘age’ such that $N_{i}(0)>1$ for some *i*. Another assumption that could be relaxed is that each individual produces descendants with an identical offspring distribution as specified by the lineage. Thus, relaxing this assumption allows the birth rate within a lineage to fluctuate along specific branches of that lineage over time, which would be useful if, for example, an infected CD4+ T-cell differentiates into another CD4+ T-cell phenotype with a distinct birth rate. The derived model is known as a multi-type branching process (Conway and Coombs 2011; Nordon et al. 2011). Individuals are classified into *m* types such that the number of offspring is a vector $y=[y_{1}\ldots y_{m}]$ where each *y_j_* is a natural number (including zero) that represents the number of offspring of each type. The probability that an individual of type *j* has the offspring vector ***y*** is given by $p_{j}(y)$. Finally, a third assumption that would be useful to relax is that of time-homogeneous offspring distributions, also known as a branching process under varying environments (see Mitov and Omey 2014), such that the offspring distributions of individuals change with time.

### 3.3 Homogeneous impact of proliferation

Once the impact of proliferation on persistence has been established, this relationship must be assessed by multiple experiments and its consistency verified. This can currently also be done by considering the values of *p*_1_ or *p*_2_ across the myriad of studies that have already used these summary statistics. However, despite the widespread application of *p*_1_ or *p*_2_ to investigate clonal expansion, studies lack consistency when aggregating these proportions across multiple time points and individuals. For example, some have reported these statistics for each participant’s time-points separately (Maldarelli et al. 2014; Wagner et al. 2014; Von Stockenstrom et al. 2015; Lorenzi et al. 2016; Salantes et al. 2018), while others have aggregated the sequence data from multiple participants, with a single time-point each, and reported these statistics for the entire data set (Maldarelli et al. 2014; Hosmane et al. 2017; Lee et al. 2017). These inconsistencies in reporting hinder objective comparison of the results.

### 3.4 Null model

The disproportionate abundance of one or more sequence variants has caused many researchers to speculate on whether there is a mechanistic basis that causes certain reservoir sub-populations to be more prone to clonal expansion. Since the null model provides the expected outcome if chance alone is responsible, it can be used to detect whether the process in question displays non-trivial features in the data. Therefore, the null model, where proliferation rates are homogeneous among reservoir lineages, would oppose the proposed mechanistic basis for rate variation among reservoir lineages. This null model is plausible if cell division is indiscriminately governed by homeostatic proliferation or nonspecific immune activators. Moreover, the skewed distribution of abundance across sequence variant labels (Figure 1B) can be explained by this null model, which we can express as:

$$\lambda =\lambda_{i}=\lambda_{k}\;\forall i,k\in V(t)$$

where *λ* is the lineage-independent birth rate such that the expected number of offspring $\Lambda =\int_{0}^{t}\lambda (t)dt$. To explain, consider the branching process where proliferation rates are equivalent (lineage-independent) among reservoir lineages. For simplicity assume, as before, that each individual lives for a single unit of time and that offspring are generated at the same time such that there are discrete generations over time. The number of offspring *y* produced per individual could follow a geometric distribution, for example, that is dependant on the probability that no offspring are produced $p=\frac{1}{1+\Lambda }$:

$$P(Y|p)={\left(1-p\right)}^{y}p.$$

Given this offspring distribution, the abundance of a lineage at generation time *t* depends on both the abundance of the lineage in the previous generation $\left(N_{i}(t-1)\right)$, and the offspring distribution:

$$N_{i}(t)=\sum_{j=1}^{N_{i}(t-1)}Y_{j}.$$

Stochasticity in the number of offspring per individual implies that despite the inherent similarity between lineages the particular lineage with the highest abundance is a random outcome. In the above example, the requirement that each individual live only for a single time unit can be relaxed such that the death rate is effectively reduced and μ<λ ∀i, where *μ* and *λ* are the lineage-independent death and birth rates. Under this relaxed assumption the only difference will be the number of generations required until a dominant lineage emerges which will be governed by the magnitude of the net-rate (λ−μ). Nevertheless, the lineage with the highest abundance will be a random outcome of the process. Under this null model, the mere existence of clones is not an adequate criterion for reaching a conclusion regarding the contribution of cell-heterogeneous proliferation, instead numeric estimates of the birth rates are required.

### 3.5 Mechanistic basis for rate variation

In practice, there is no explicit distinction made between the potential stochastic and deterministic components of within-host reservoir populations, and generally highly abundant sequence variants are considered to be due to some predetermined feature of the infected cell or HIV-1 infection. This can be mathematically stated as:

$$\exists i\;s.t.\;\lambda_{i}>\lambda_{k},\;i\neq k$$

where *λ_i_* is the lineage-dependent birth rate. For example, the CD4+ T-cell phenotype (Lee et al., 2017), its antigen-specificity (Simonetti et al., 2016), and the integration site of HIV-1 (both the particular gene (Maldarelli et al. 2014; Haworth et al. 2018) and its biological pathway (Haworth et al. 2018)) are all possible features that could drive distinct birth rates between lineages and are considered in turn below.

Cell sorting studies illuminate the various CD4+ T-cell phenotypes that contain HIV-1 DNA, and while it is widely accepted that CD4+ T cells of a memory phenotype harbor a substantial proportion of the HIV-1 reservoir, sub-categories among these infected cells have been identified (Murray et al. 2016; Hiener et al. 2017; Lee et al. 2017), including transition memory (TM), central memory (CM) and effector memory (EM) T cells. Additionally, a less differentiated stem-cell (SM) type may also persist during long-term treatment (Buzon et al. 2014; Von Stockenstrom et al. 2015). The outcomes of cell sorting studies can theoretically be represented by the vectors $\lambda =[\lambda_{1}\ldots \lambda_{n}]$ and $\mu =[\mu_{1}\ldots \mu_{n}]$, where *λ_i_* and *μ_i_* are the lineage-dependent birth and death rates. The number of lineages *n* depends on the lineage specification used by each study. Subsequently, a study investigating distinct proliferation and/or decay rates between the TM, CM, EM and SM phenotypes would hypothesize that:

Var(λ)>0 or Var(μ)>0, where i∈{TM,CM,EM and SM}.

Interestingly, variations in these rates may shift the relative contribution of each phenotype to the reservoir over time. For example, EM cells that have comparatively high proliferation rates may constitute the largest proportion of the reservoir during early treatment (Buzon et al. 2014). However, since EM cells decay quickly, CM cells that have comparatively lower proliferation and decay rates may eventually surpass the EM cell population to comprise the largest proportion of the reservoir (Buzon et al. 2014). The specification of distinct lineages *i* over which *λ_i_* and *μ_i_* are valid can be a challenge, since cell phenotypes may change over time leading to the migration of sequence variants between the distinct cell phenotypes (Von Stockenstrom et al. 2015; Hiener et al. 2017), confounding the results if not accounted for. Nevertheless, further cell sorting studies will not only illuminate the various cell phenotypes that contain HIV-1 DNA but also their dynamics in infected individuals during treatment.

Another predetermined cell feature that could drive proliferation is antigenic stimulation by specific, common antigens. An example of this was demonstrated by Simonetti et al. (2016) who observed extensive clonal expansion in the presence of cancer metastases, suggesting that an infected cells harboring a replication-competent sequence variant proliferated in response to a cancer antigen. Similarly, others have hypothesized that a chronic state of immune activation, caused by the continuous activation of infected cells leading to the release HIV-1 antigens, drives the clonal expansion of HIV-1 targeting reservoir cells (Mann et al. 2020). Advances in the characterization of T-cell receptors will be critical for understanding the role of antigen-driven clonal expansion on reservoir persistence.

While the features discussed above were governed by the interaction of the infected cell with its environment, some have speculated that HIV-1 infection could directly play a part in persistence by way of the integration site. Maldarelli et al. (2014) showed that proviruses integrated in the BACH2 and MKL2 genes, which are thought to be involved in the growth and development of cells, had distinct characteristics compared to control experiments of acute infection *in vitro*, implying an advantage for proviruses with these integration sites (Maldarelli et al. 2014). Specifically, these proviruses were in the same orientation as the host genes and highly restricted to a specific region of the BACH2 and MKL2 genes for a participant on long-term ART treatment, whereas no such pattern in the distribution of HIV-1 integration sites were observed in the controls (Maldarelli et al. 2014). While this pattern is unusual, the exact mechanism by which it benefits persistence was not considered; however a mechanism that increased the birth rate of these lineages was implied. It has yet to be established if the relationships between the sequence variant abundance and CD4+ T-cell phenotype, antigen-specificity or integration site are present in a substantial number of cases. In fact, the sampling bias toward sequence variants with large abundances driven by alternative mechanisms in distinct infected individuals may hinder such studies. For example, if there are two distinct drivers of clonal expansion operating in a single individual the driver responsible for the sequence variant with the highest abundance may mask the effect of the other driver, since samples will be biased toward the highest abundance driver at the time of sampling.

In summation, we contend that summary statistics of clonality such as the proportions *p*_1_ and *p*_2_ are not up to the task of assessing the underlying hypotheses of most reservoir studies, which requires estimation of lineage-specific birth and/or death rates in the reservoir. While these statistics have contributed to our understanding of the HIV-1 reservoir, we have identified several limitations in these quantities. Ultimately, the choice of the most appropriate measurement depends on the experimental question that is being asked, but birth and death rate estimates of reservoir lineages will likely provide a useful representation of the underlying hypothesis. Further, articulating the hypothesis in terms of variation in birth/death rates links the problem to a rich theoretical literature on branching processes. While the proportional measurements *p*_1_ and *p*_2_ do not take lineage-heterogeneity or time-heterogeneity into account, the birth and death rate estimates can be easily extended to include both.

### 3.6 Impact of time heterogeneity

Thus far, we have discussed rates of expansion and decay in the latent reservoir as though they are constant over time. There is growing empirical evidence that the abundance of clones in the reservoir varies substantially over time. For example, Wang et al. (2018a) observed that some HIV-1 sequence variants in the latent reservoir were abundant in some samples and absent from others, while other variants persisted at consistent levels over a time scale of years. Such results allude to more complex dynamics underlying the abundance of a sequence variant $N_{i}(t)$ over time than can be explained by constant rates of growth/decay, or limited stochastic variation around these rates over time. However, these observations do not provide conclusive proof of time-heterogeneity in growth and/or decay rates in the reservoir because they do not account for incomplete sampling or ambiguous labeling, as discussed in the preceding sections. This motivates a detailed investigation of time-heterogeneity in instantaneous lineage-specific birth and death rates that cannot be directly observed, i.e. $\lambda_{i}(t)$ and $\mu_{i}(t)$, based on the variation of estimable quantities over time such as $N_{i}(t)$.

Quantifying the time-heterogeneity of growth and decay rates in the reservoir is complicated by the possibility of rate variation among virus lineages in association with, for example, CD4+ T-cell phenotype, integration site, or antigen-specific responses. This situation is similar to the ‘selection inference uncertainty principle’ encountered by branch-site models of episodic selection (Murrell et al. 2012), where it is not feasible to parameterize a full model of non-synonymous substitution rate variation among sites and over time at the highest granularity—*viz.*, an independent rate parameter for every combination of codon site in the alignment and branch in the tree. To distinguish between different hypotheses of rate heterogeneity, it is necessary to introduce some additional mathematical notation. We denote the expected birth rate of the *i*-th lineage at time *t* as a deviation from the grand mean (*α*):

$$\lambda_{i}(t)=\alpha +\beta_{i}+f_{i}(t)$$

where *β_i_* is the time invariant lineage-specific effect, and $f_{i}(t)$ is some function that represents the lineage-specific heterogeneity over time. Note that all lineages may share the same time-heterogeneous trend in rates such that $f_{i}(t)=f(t)\;\forall \;i$. Although it is conceivable that $f_{i}(t)$ could assume the form of any smooth continuous function, it would be exceedingly difficult to parameterize this smooth function from experimental data. Instead, it could be more feasible to fit a linear function to incorporate the transient effects of measurable quantities, such as co-infection (see below). We can apply a similar approach to model variation in death rates among lineages and over time, i.e. $\mu_{i}(t)$.

Let us assume that the partially observable quantities $N_{i}(t)$ and $V_{i}(t)$ are stochastic processes that are shaped by independent realizations of $\lambda_{i}(t)$ and $\mu_{i}(t)$ over time. For example, the hypothesis that variants are identical with respect to expected birth rates that may vary over time corresponds to constraining $\beta_{i}=0$ and $f_{i}(t)=f(t)$ for all *i*. Under these conditions, stochastic variation in the lineage birth process will cause some lineages to become more abundant than others over time. The identity of the most abundant lineages would be a random outcome. On the other hand, we expect a specific set of lineages *S* to be reproducibly more abundant if $\beta_{i}>0$ for i∈S and/or $\beta_{j}<0$ for j∉S and $f_{i}(t)=0\;\forall \;t$. Identifying these deterministic effects is difficult because it requires the experimental replication of the growth process from identical starting conditions, which is difficult to attain *in vitro* and not feasible *in vivo*. It would be more feasible to identify characteristics that are deterministically associated with variation in *β_i_*, such as the occurrence of integration sites in a predefined subset of genes (Maldarelli et al. 2014). This parameter-rich approach to modeling within-host dynamics is what would be required to formally test the hypotheses that have been described verbally in the literature.

For example, Wang et al. (2018a) concluded that the observed patterns of waxing and waning frequencies was not consistent with the sustained expansion of specific lineages, i.e. $\lambda_{i}(t)>\mu_{i}(t)\forall t$ for some subset of lineages indexed by *i*. Since variation in proliferation rates driven by the location of integration sites would likely result in a more consistent pattern of clonal expansion, these data suggests that other drivers such as antigen stimulation may have played a greater role. However, it is debatable whether or not integration site-driven expansion is sustained over time. If this effect is an outcome of HIV-host spliced genes (Maldarelli et al. 2014; Pinzone et al. 2019), for example, then some external stimulation would potentially be required, for example via antigen stimulation or shock-and-kill treatment (Pinzone et al. 2019). The difference between integration site-promoted expansion and other drivers or cells not subject to such effects may be detected in the instantaneous rates λ(t) and μ(t) or their respective dynamics. For example, once externally stimulated, a cell with an integration site that promotes its expansion will achieve a higher maximum abundance than a similar cell without the integration site that promotes expansion i.e. $\max(N_{\text{integration}\;\text{site}})>\max(N_{i})$, where *i* represents lineages with an alternative integration site or cell lineages that are uninfected. Since this maximum abundance is governed by the instantaneous lineage birth and death rates prior to the emergence of the maximum, estimating these rates across multiple samples may expose differences between drives or distinct lineages. To summarize, neither the qualitative measures describing the pattern in the abundance of sequence variants nor the proportions *p*_1_ and *p*_2_—which do not include a time component—are sufficient for the rigorous assessment of these alternative hypotheses. Moreover, these additional caveats should be noted when considering both our and their conclusions: some samples did not exhibit a waxing and waning pattern (Wang et al. 2018a); different sampling frequencies could have different patterns; the impact of under-sampling was not accounted for; and defining lineages by attributes (discussed below) other than, or in addition to, the sequence variant may be required.

The current literature exclusively uses the integrated HIV-1 sequence variant or integration site to define lineages, i.e. each *i* represents a sequence variant over which *λ_i_* and *μ_i_* is defined. While potential alternative definitions have been alluded to, for example the CD4+ T-cell phenotype or the general features of the HIV-1 provirus in the host gene (provirus orientation, sub-gene regions, *etc.*), the question regarding the usefulness of such alternative definitions still remains untested. Furthermore, if alternative definitions of lineages are used, should these define additional lineages of each sequence variant if the definition is dynamic (e.g. cell phenotypes that can change over time (Von Stockenstrom et al. 2015; Hiener et al. 2017)) or replace the current strategy of using the sequence variant to define a lineage? The primary reason for considering an alternative definition of a lineage is to test the hypothesis that substantial rate variation exists. However, alternative definitions may also be useful in obtaining more precise parameter estimates, e.g., *β_i_* and $f_{i}(t)$, and combining data from different sources. If lineages are defined by the cell phenotype, for example, sequence variants that share this phenotype can be combined to estimate their hyper-parameters; or if variation among replication-competent and non-competent proviruses is more pronounced in their death rates, proviruses could be grouped accordingly. However, given the uncertainty surrounding alternative lineage definitions we propose that HIV-1 reservoir data measurements be reported as proliferation and decay rates for the observed sequence variants for longitudinal samples, i.e. $\lambda_{i}(t)$ and $\mu_{i}(t)$, estimated from sequence variation or phylogenies and variation in these, i.e. $\mathrm{Var}(\lambda_{i}(t))$ or $\mathrm{Var}(\mu_{i}(t))$, when lineage specific rates are under consideration. It is our hope that these measurements, along with longitudinal and characterization studies, will shed light on both the emerging pattern, created by varying proliferation and decay rates, and the cause of variation.

Finally, we have not discussed the problem of determining when the lineages in the reservoir were seeded. Similar to the potential time-heterogeneity in the lineage birth and death rates, this research question may be complicated by similar heterogeneity in the rates that cells transition (migrate) between active (*A*) and latently-infected (*L*) states. Specifically, at some time-point *t_i_* the rate at which lineages moved to the latent state may have been higher than at another time-point *t_j_*—i.e. $m^{A\to L}(t_{i})>m^{A\to L}(t_{j}),\;i\neq j$. For example, a high viral load may increase the probability that resting T cells become infected; alternatively, the probability that infected cells enter a latent state may increase following treatment initiation and immune restoration. Regrettably, it is difficult to directly investigate reservoir seeding rates ($m^{A\to L}(t_{i})$) during active infection since most sampled cell populations represent active infections. Therefore, we rely on retrospective phylogenetic studies that estimate when lineages were first integrated into the reservoir by comparing their proviral sequences to the circulating genetic variation before treatment, e.g. (Brodin et al. 2016; Jones et al. 2018; Abrahams et al. 2019). Regardless of the dating methodology employed, these studies also rely on sampling extant variants from the reservoir, such that lineages with higher rates of proliferation are over-represented. Thus, whether or not these estimates can yield an accurate picture of the migration rates $m^{A\to L}$ will depend on the drivers that govern clonal expansion. For example, a driver that instantaneously induces proliferation for a random subset of lineages in the reservoir at the same exact moment will be more likely to result in a representative sample, and consequently better estimates of migration rates, than a deterministic driver or a more complicated time-heterogeneous driver. Estimates of integration dates could also provide context for the observed lineage proliferation/decay rates for the first longitudinal sample, or if only one sample is available. With this context, the proliferation/decay rates can be more readily compared between lineages and across multiple sampled time-points.

## 4. Conclusion

The latent viral reservoir is a key barrier to a curing HIV-1; however, measuring the reservoir and its composition robustly is still a challenge for the field. While quantifying the clonality of the reservoir has increased our understanding of the reservoir, the most frequently used proportional statistics have several limitations that need to be addressed. These limitations arise, in part, due to the fact that the observed frequencies of labels in samples is an emergent property of the underlying within-host population—the state of which is largely unknown and potentially time-heterogeneous. Given these factors we propose that the birth and death rates be estimated for distinct reservoir lineages, by using statistical and phylogenetic models. In addition, we propose that, depending on the context, the birth and death rates be either directly related to persistence or the variation between lineages calculated. Combining birth and death rate estimates with longitudinal samples and new sequencing strategies will facilitate better characterization and monitoring of proviral dynamics. With improving access to effective ART, viral suppression is being achieved by a growing proportion of people living with HIV-1. As a result, the longitudinal samples of untreated chronic infections that have historically driven phylogenetic studies of HIV-1 evolution within hosts are increasingly scarce. Modeling the dynamics of the latent viral reservoir is the next frontier in the study of HIV-1 within-host evolution and phylodynamics, where we must adapt existing models and/or develop new models to support the development of cure/eradication strategies.

## Funding

This work was supported by a Project Grant from the Canadian Institutes of Health Research (PJT-155990), and in part by the Division of Intramural Research, National Institute of Allergy and Infectious Diseases (NIAID), National Institutes of Health (NIH).

**Conflict of interest:** None declared.
